# Electronic excitation spectra of organic semiconductor/ionic liquid interface by electrochemical attenuated total reflectance spectroscopy

**DOI:** 10.1038/s42004-021-00525-y

**Published:** 2021-06-11

**Authors:** Ichiro Tanabe, Iroha Imoto, Daijiro Okaue, Masaya Imai, Shohei Kumagai, Tatsuyuki Makita, Masato Mitani, Toshihiro Okamoto, Jun Takeya, Ken-ichi Fukui

**Affiliations:** 1 Graduate School of Engineering Science, Osaka University, Osaka, Japan; 2 Graduate School of Frontier Sciences, The University of Tokyo, Chiba, Japan; 3 Department of Photomolecular Science, Institute for Molecular Science, Myodaiji, Okazaki, Aichi, Japan

**Keywords:** Surface spectroscopy, Electronic devices, Analytical chemistry

## Abstract

The interface of organic semiconductor films is of particular importance with respect to various electrochemical devices such as transistors and solar cells. In this study, we developed a new spectroscopic system, namely electrochemical attenuated total reflectance ultraviolet (EC-ATR-UV) spectroscopy, which can access the interfacial area. Ionic liquid-gated organic field-effect transistors (IL-gated OFETs) were successfully fabricated on the ATR prism. Spectral changes of the organic semiconductor were then investigated in relation to the gate voltage application and IL species, and the magnitude of spectral changes was found to correlate positively with the drain current. Additionally, the Stark shifts of not only the organic semiconductor, but also of the IL on the organic semiconductor films were detected. This new method can be applied to other electrochemical devices such as organic thin film solar cells, in which the interfacial region is crucial to their functioning.

## Abstract (web summary)

Interfaces are a key functional element in organic devices, but accessing buried interfaces has been challenging, Here the authors present a novel spectroscopic setup to investigate the spectral properties of ionic liquid/organic films interfaces under an electric field.

## Introduction

Organic field-effect transistors (OFETs) have attracted much attention as they are light in weight, flexible, low cost, and can be easily processed^1,2^. In particular, electric double-layer OFETs (EDL-OFETs) have been intensively investigated because of their significantly low operation voltage (<1 V) compared to conventional SiO_2_-gated OFETs (>10 V)^3–6^. In EDL-OFETs, a high electric field is generated in the EDL that accumulates in the interfacial region between the organic semiconductor and electrolyte, resulting in a low operation voltage. Therefore, the organic semiconductor/electrolyte interface is especially important.

Ionic liquids (ILs) are promising materials as novel electrolytes because of their extremely low vapor pressure, high thermal stability, and wide potential window^7,8^. IL-gated EDL-OFETs were first reported by Takeya’s group in 2008 using rubrene single crystals^9^. This field of study has since expanded to include other organic semiconductors, such as octathio[8]circulene^10^, [7]phenacene^11^, pentacene^12–14^, and fullerene^13^. The IL species is one of several important factors determining the carrier mobility (*μ*) of a device^15–17^. This was demonstrated in a study by Fujimoto et al.^16^ in which six kinds of ILs were used as the electrolyte for the octathio[8]circulene EDL-OTFTs. Based on these results, it was proposed that the carrier mobility in organic semiconductors is affected by the polarizabilities and sizes of the anions of the ILs. In another example, Ono and co-workers compared the carrier mobilities of rubrene single crystals and 7,7,8,8-tetracyanoquinodimethane, using five IL species. They found that the lower capacitance of the IL led to the higher carrier mobility, and concluded that the coupling between the mobile carrier and ILs was the key parameter for efficient carrier transport^17^.

In this context, information regarding the interactions between organic semiconductors and ILs is crucial in gaining a better understanding of the mechanism of high-performance EDL-OFETs. However, experimental techniques that can access the organic semiconductor/electrolyte interface are limited. Interface-sensitive spectroscopic methods such as second harmonic generation (SHG)^18^ and sum-frequency generation (SFG)^19^ have been applied to organic semiconductor/gate dielectric (SiO_2_) interfaces. Attenuated total internal reflection Fourier transform infrared (ATR-FTIR) spectroscopy has been used to investigate organic semiconductor/polymer electrolyte dielectric interfaces^20^. Recently, operand X-ray nano-spectroscopy was also applied to the EDL-OFETs^21^. These previous studies reveal conformation and electronic state changes of organic semiconductors as a result of applied voltages. However, so far, the previous studies focused mainly on organic semiconductors. The EDL-OFETs express their functions due to the interaction between the organic semiconductor and the electrolyte, and thus, the interfacial electrolyte on the semiconductor surface also should be investigated.

In this study, we propose an original, electrochemical ATR-UV spectroscopic method for investigating the organic semiconductor/IL interface of the EDL-OFET. In the UV region, the penetration depth, which corresponds to the light path length, is less than 50 nm. Therefore, surface-sensitive measurements can be achieved. In addition, many kinds of electrolytes have strong absorbance in the UV region, and thus, the absorbance of not only the organic semiconductor but also the IL can be investigated. Three,11-dinonyldinaphtho[2,3-*d*:2′,3′-*d*′]benzo[1,2-*b*:4,5-*b*′]dithiophene (C_9_-DNBDT-NW) was used as an organic semiconductor, and 1-ethyl-3-methylimidazolium bis(fluorosulfonyl)amide ([EMIM][FSA]) and *N,N,N*-trimethyl-*N*-propylammonium bis(trifluoromethanesulfonyl)amide ([TMPA][TFSA]) were used as the ILs. Upon applying a voltage, the absorption intensity and wavelength of the organic semiconductor were decreased and blue-shifted, respectively. These results represented carrier (i.e., hole) injection into the organic semiconductor, and the Stark shift was induced by the electric field generated in the organic semiconductor/IL interface. The wavelength shift width depended on the IL species, indicating a difference in the electrostatic interaction between the organic semiconductor and ILs. A similar relationship between the injected carriers and the spectral changes was reported by other optical methods such as ATR-FTIR and SFG spectroscopies^19,20^. In addition, the electronic absorbance and Stark shifts of both the semiconductor and the IL were successfully measured. As a result, IL-dependent spectral changes were revealed, which indicates the different degrees of interactions between the IL species and the organic semiconductor surface. Such a direct observation of interfacial ILs is a unique advantage of the ATR-UV spectroscopic technique presented herein.

## Results

### Transmission spectra of C_9_-DNBDT-NW solution and film

**Fig. 1:**
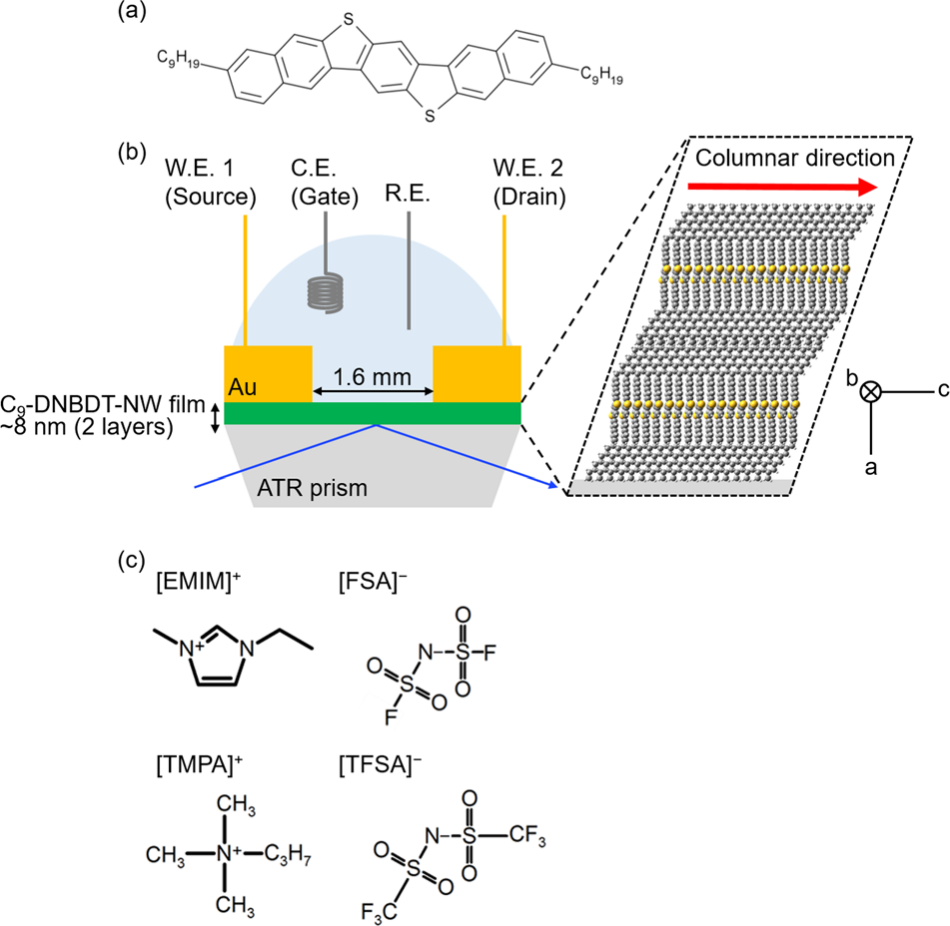
A developed electrochemical ATR-UV spectroscopy for ILs-gated EDL-OFETs. **a** The structural formula of C_9_-DNBDT-NW. **b** A schematic illustration of the fabricated ILs-gated EDL-OFET on the ATR sapphire prism. **c** The structural formulae of ILs employed in this study.

We developed a new electrochemical ATR-UV (EC-ATR-UV) system. The structural formula of C_9_-DNBDT-NW and the schematic illustration of the fabricated device are shown in Fig. 1a, b, respectively. According to the reported procedure^22,23^, single crystal-like thin films with two molecular layers of C_9_-DNBDT-NW were prepared on the ATR prism. Commercially available [EMIM][FSA] and [TMPA][TFSA] were employed as the ILs (Fig. 1c).

**Fig. 2:**
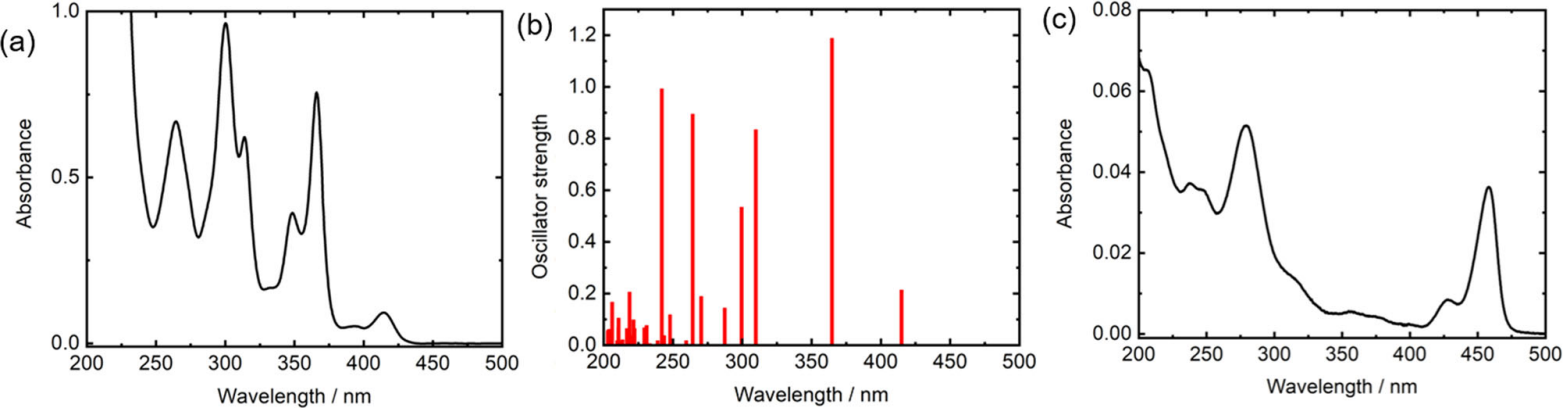
Spectroscopy and TD-DFT calculation of C_9_-DNBDT-NW molecule and film. **a** A transmission spectrum of the C_9_-DNBDT-NW/dichloromethane solution (100 μM). **b** Calculated vertical transitions of a C_9_-DNBDT-NW molecule. **c** A transmission spectrum of the C_9_-DNBDT-NW film on a sapphire substrate.

Figure 2a shows the transmission spectrum of the C_9_-DNBDT-NW/dichloromethane solution (~100 μM). The strong absorbance below 250 nm was attributed to the dichloromethane solvent; the solvent had no absorbance in the wavelength region above 250 nm. The spectrum exhibited some sharp peaks at 415, 366, 349, 314, 300, and 264 nm, and small peaks at ~390 and ~330 nm. According to the time-dependent density functional theory (TD-DFT) calculation (Fig. 2b), the calculated absorbance at 414.9 nm is due to electronic excitation, primarily from the highest occupied molecular orbital to the lowest unoccupied molecular orbital. The calculated vertical transitions and their main assignments are summarized in Table S1 and Fig. S1. The clearly observed absorbance at ~349 nm in Fig. 2a was not simulated in Fig. 2b. This is due to the spin–orbit coupling of sulfur^24^, which was not incorporated in this calculation. This effect similarly accounts for other peak differences between the experimental observations and the simulations, e.g., small peaks at ~390 and ~330 nm only appeared in the experimental results.

Figure 2c shows a transmission spectrum of the C_9_-DNBDT-NW film on a sapphire substrate. Compared to the spectrum of the dichloromethane solution (Fig. 2a), the first excitation absorbance is observed at a longer wavelength (~455 nm), which is due to π stacking. Another notable difference pertains to the significantly weaker absorbance peak in the spectrum of the film at ~350 nm. This is due to the molecular orientation on the substrate as discussed in Fig. S2. Briefly, the electronic transitions along the *y*-axis primarily contribute to the obtained spectrum. This is because the *x*-axis of C_9_-DNBDT-NW is perpendicular to the substrate, which means that the electric field direction of the incident light is also perpendicular to the x-axis and thus, the transition strength along the *z*-axis is small.

### Gate voltage dependence of ATR spectra of C_9_-DNBDT-NW films

**Fig. 3:**
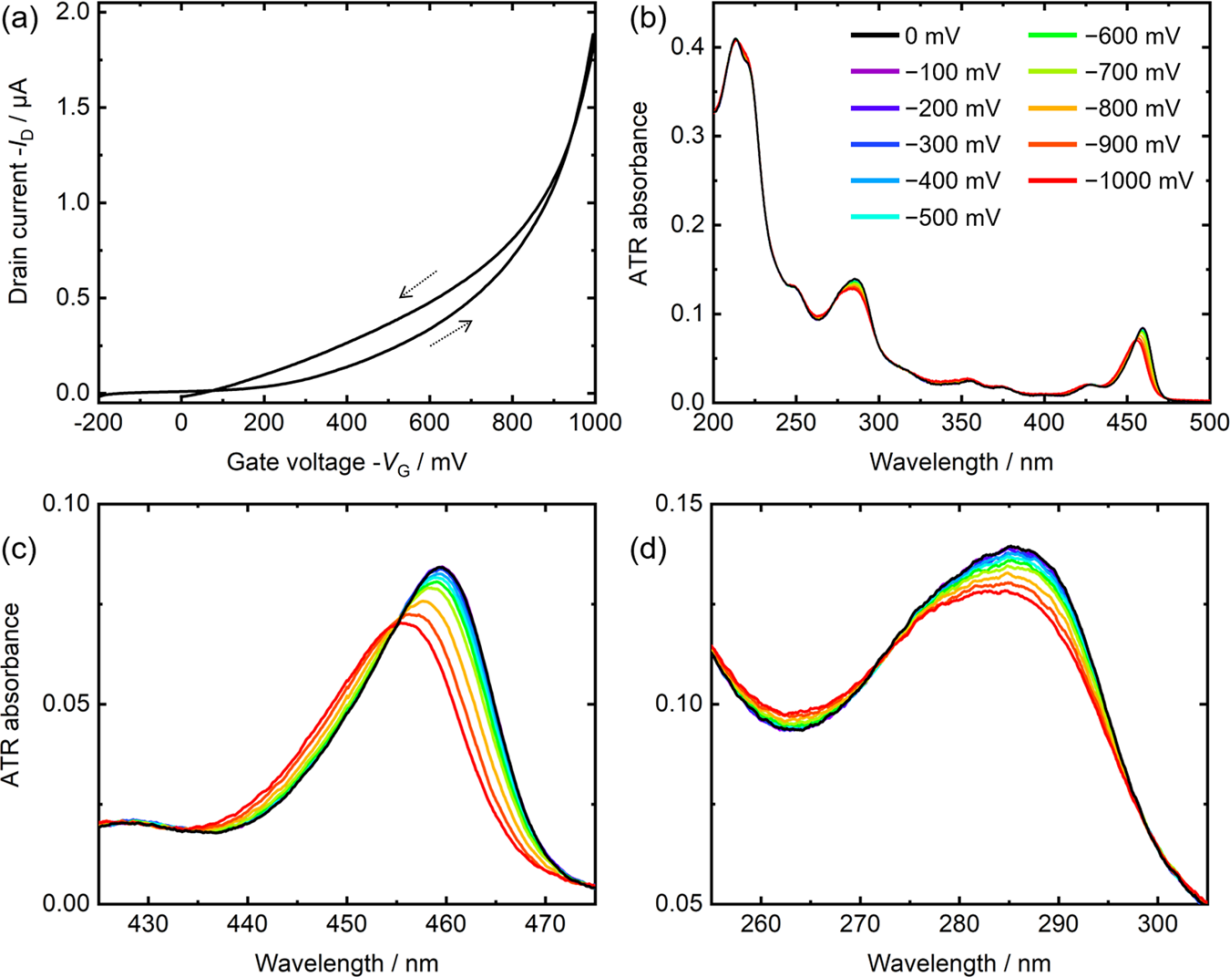
Electrochemical behavior of a C_9_-DNBDT-NW film with [EMIM][FSA]. **a** A transfer curve of the fabricated device measured at a drain voltage *V*_D_ = −100 mV and scan rate = 20 mV s^−^^1^. **b** ATR spectra of a C_9_-DNBDT-NW film with [EMIM][FSA] on a sapphire substrate. Enlarged spectra in (**c**) 425–475 nm and **d** 255–305 nm, at different gate voltages.

The fabricated device described in Fig. 1 worked successfully as the transistor at a drain voltage *V*_D_ = −100 mV and a scan rate = 20 mV s^−1^, as shown in Fig. 3a. It is worth noting that this is the first report of an IL-gated EDL-OFET using a DNBDT-based organic semiconductor. The estimated mobility was approximately 5 cm^2^ V^−1^ s^−1^, which is a reasonable value as discussed in Section S1 in the Supplementary Materials. Figure 3b shows the ATR spectrum of the C_9_-DNBDT-NW film with the IL. It has distinct peaks at ~460 and ~285 nm, similar to the transmission spectrum in Fig. 2c. The strong absorbance at ~220 nm is due to the IL on the C_9_-DNBDT-NW film; this is discussed later in the paper. By applying the gate voltage, the peaks at ~460 nm (Fig. 3c) and ~285 nm (Fig. 3d) were shifted to shorter wavelengths, and their spectral intensities decreased. It should be noted here that these spectral changes were not due to the dissolution of the organic semiconductor molecule into the IL. While other organic semiconductors such as rubrene were easily dissolved into ILs^14^, the DNBST-based OFETs were stable in ILs.

### Quantity relationship between spectral changes and charge injections

**Fig. 4:**
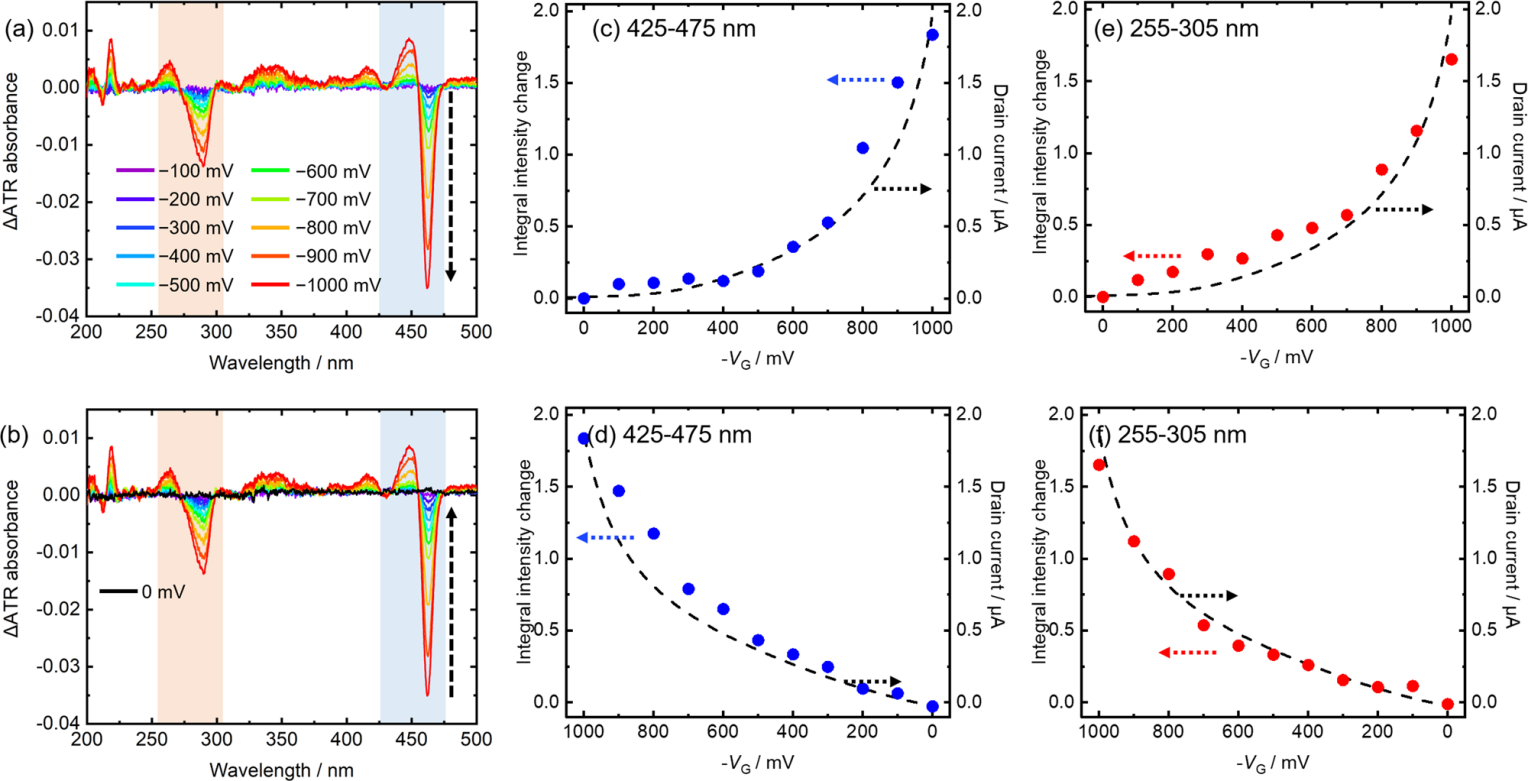
Relationship between spectral behavior and device performance. **a**, **b** Difference spectra of a C_9_-DNBDT-NW film and **c**–**f** their integral intensity changes as a function of the applied voltage in the **c**, **d** 425–475 nm and **e**, **f** 255–305 nm regions under gate voltage application (**a**, **c**, **e**) from 0 to −1000 mV, and (**b**, **d**, **f**) from −1000 to 0 mV. Arrows in **c**–**f** show the axis to see.

Figure 4a, b shows the difference spectra of the C_9_-DNBDT-NW film before and after the applied voltage. As discussed later, the spectral change was reversible, and there was little hysteresis. In order to quantify the spectral changes, the integral intensity changes in the 425–475 nm (Fig. 4c, d) and 255–305 nm (Fig. 4e, f) regions were plotted as a function of the applied voltage. The dashed line represents the drain current. A strong positive relationship was observed between the magnitude of spectral changes and the current densities. This was attributed to the decrease in the electron density in the C_9_-DNBDT-NW molecules upon carrier (i.e., hole) injection, resulting in spectral bleaching. Indeed, by the charge modulation spectroscopy (CMS) in the IR and UV regions, the positive relationship between the magnitude of spectral changes and the drain current was reported for OFETs using *N-N*′-dioctyl-3,4,9,10-perylene tetracarboxylic diimide^20^, pentacene^25^, and rubrene^26^. On the other, as discussed later, the absorbance and its gate voltage dependence of the ILs were presented, for the first time, by using the EC-ATR-UV spectroscopy.

### IL dependence on the spectral changes

**Fig. 5:**
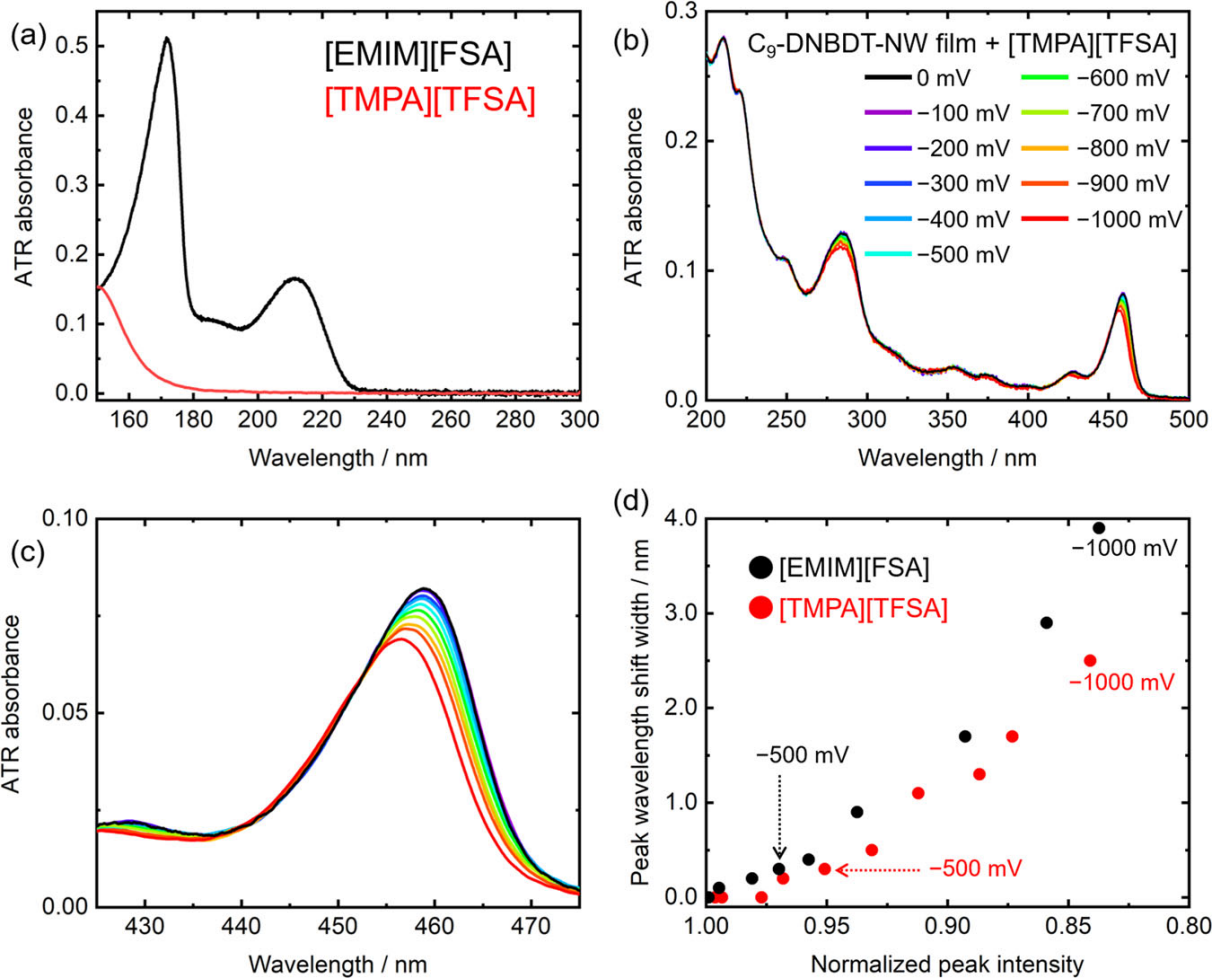
Effects of IL species on the EC-ATR-UV spectra. **a** ATR spectra of [EMIM][FSA] and [TMPA][TFSA]. **b** ATR spectra of a C_9_-DNBDT-NW film with [TMPA][TFSA]. **c** Enlarged spectra in the 425–475 nm region. **d** Relationship between peak intensities (425–475 nm) and wavelength shift width of the C_9_-DNBDT-NW film with [EMIM][FSA] and [TMPA][TFSA].

Subsequently, IL dependence of the spectral changes of the C_9_-DNBDT-NW film was investigated. The IL [EMIM][FSA] was replaced with [TMPA][TFSA] as the gating electrolyte. ATR spectra of these ILs are shown in Fig. 5a. While [EMIM][FSA] has a characteristic absorbance around 210 nm, [TMPA][TFSA] has no absorbance above 200 nm. The fabricated device with [TMPA][TFSA] (Fig. 5b) displayed the same spectral behaviors as that with [EMIM][FSA] (Fig. 3b): the peak wavelengths and intensities were blue-shifted and decreased in the 425–475 nm (Fig. 5c) and the 255–305 nm regions.

As will be discussed in the Discussion section, a peak wavelength shift was observed due to the change in molecular polarizability. A comparison of the two ionic species, summarized in Fig. 5d, addresses the relationship between the peak wavelength and the peak intensity at various applied gate voltages. Interestingly, the shift width was significantly different between the two devices employing different ILs. In contrast, the extent of spectral decreasing was similar at the gate voltage *V*_G_ = −1000 mV.

### Spectral behavior of ILs

**Fig. 6:**
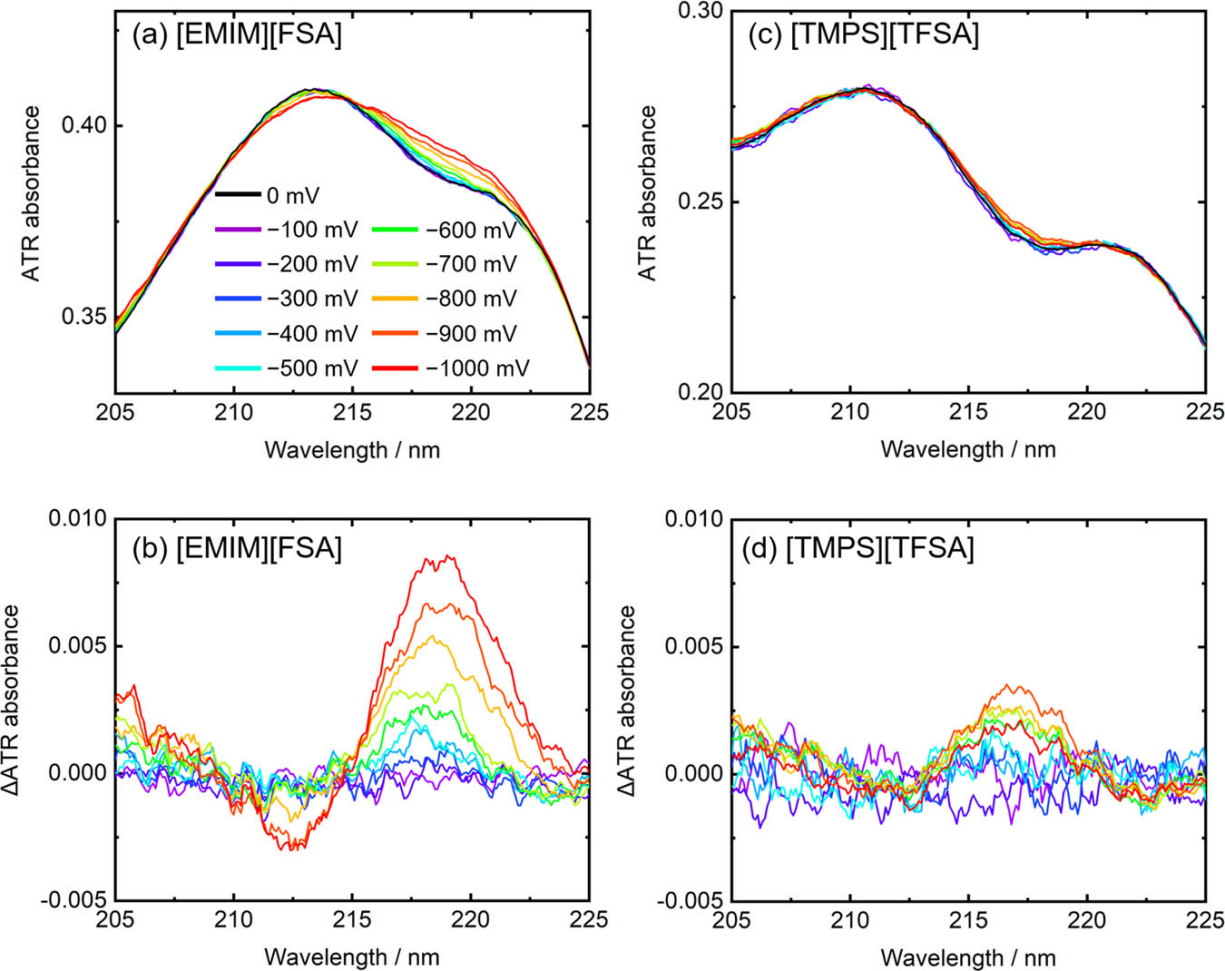
Spectral changes of ILs. **a**, **c** ATR and **b**, **d** difference spectra of C_9_-DNBDT-NW films in the 205–225 nm region with **a**, **b** [EMIM][FSA] and **c**, **d** [TMPA][TFSA]. While [EMIM][FSA] has absorbance, [TMPA][TFSA] has no absorbance in the 205–225 nm region.

Finally, let us consider the wavelength region between 205 and 225 nm. As described above (Fig. 5a), [EMIM][FSA] had a strong absorbance in this region. The absorbance around 210 nm of imidazolium-based ILs with fluorine anions is due to the inner molecular excitation of the imidazolium cation^27^. Figure 6a, b shows that upon the voltage application, the absorbance around 212 nm decreased and that around 218 nm increased, which indicates that the absorbance of [EMIM][FSA] was red-shifted. When [TMPA][TFSA] was employed as the IL, only a slight spectral change was observed upon the voltage application (Fig. 6c, d). Therefore, it can be concluded that the observed spectral change was due to the Stark shift of [EMIM][FSA] on the C_9_-DNBDT-NW film.

## Discussion

Herein, the origin of the peak wavelength shift caused by the electric field (i.e., the Stark shift) is discussed. In an external electric field ***F***, the energy levels of molecules are modulated, and the field-induced change in the transition energy (Δ*E*) between the ground and excited states is given by the following equation^28^:

1 $${\Delta}E = - {\Delta}\mu \cdot {\boldsymbol{F}} - \frac{1}{2}{\boldsymbol{F}} \cdot {\Delta}\alpha \cdot F$$

where Δ*μ* and Δ*α* are the differences in the dipole moment and molecular polarizability tensor, respectively, between the ground and excited states. Ohta’s group reported the Stark shifts of various molecules such as pyrene^29^ and rubrene^30^. The dependence of the Stark shift of OFETs on the gate voltage has been reported in the literature by several other groups^31–36^. In this study, the C_9_-DNBDT-NW molecules were fixed, and thus, the dipole moment *μ* did not change (i.e., Δ*μ* = 0). When the carriers (i.e., holes) were injected into the C_9_-DNBDT-NW film, the electric field was induced between the organic film and interfacial IL. Therefore, the observed peak shift was due to the change in the molecular polarizability of C_9_-DNBDT-NW. It should be noted here that the Stark shift of not only organic semiconductor but also IL was detected by the EC-ATR-UV spectroscopy as described later.

As shown in Fig. 5d, the relationship between the peak wavelength and the peak intensity under voltage application varied depending on the IL species. That is, [TMPA][TFSA] induced the smaller peak wavelength shift than [EMIM][FSA]. This result indicated that the interaction between the hole-injected C_9_-DNBDT-NW molecules and the ILs were varied depending on the IL species. Although the reasons for this difference have not been elucidated, several possibilities have been proposed. The first is related to the anion size of the two ILs. It is speculated that the smaller anion (i.e., [FSA]^−^) can approach the organic film surface more closely than the larger anion (i.e., [TFSA]^−^), thereby inducing a larger polarizability change. It should be noted here that negative charges of both [FSA]^−^ and [TFSA]^−^ were mainly localized at nitrogen atom (Fig. 1c), and thus, the distance of the negative charge of [TFSA]^−^ from the hole-injected OFET surface should be larger than that of [FSA]^−^. For another possible factor, it was reported that the interaction strength between organic substrates and ILs strongly depends on the species of the substrate and the IL, which have effects on microscopic structures and the dynamics of the ions near the substrate surfaces^37^. Such molecular structure and dynamics of ILs may have effects on the electrostatic interaction between the organic semiconductor substrates and ILs. In addition, the device performance of the OFETs depends on the interaction between the organic semiconductor and the dielectrics^26,38^. For example, the carrier trapping effects of OFETs are one important issue because the trapping increases the operation voltage and decreases the stability. As discussed later, the EC-ATR-UV spectroscopy can cut into the problem by utilizing its interface sensitivity. Although more experimental and theoretical investigations would be necessary to reveal the molecular dynamics at the interface, the spectroscopic dependence on the ILs is clearly shown in Fig. 5d.

In addition, it should be emphasized here that the newly developed EC-ATR-UV spectroscopy is capable of detecting the Stark shift of not only the organic semiconductor thin film but also the IL near the thin film surface as discussed in Fig. 6. The detection of the shift of the IL is difficult using the transmission spectra because of the strong absorbance of the IL as shown in Fig. S4. For example, the absorption coefficient (*ε*) of 1-butyl-3-methylimidazolium TFSI ([BMIM][TFSI]) at 211 nm is ∼5 × 10^3^ mol^−1^ dm^3^ cm^−1^ ^39^, and preparation of a thin IL film with less than ~500 nm thickness is required to obtain transmission spectra without saturation.

Another notable point in Fig. 6a is that the spectral intensity did not decrease due to the voltage application. This indicates that the cation remained close to the C_9_-DNBDT-NW surface even after the hole injection. This is one important advantage of the EC-ATR-UV spectroscopy, because such direct measurements of the interfacial ILs were achieved by the short (~50 nm) penetration depth and ILs’ strong absorbance in the UV region. The penetration depth in the present case is estimated in Section S2. According to reported MD simulations^38,40^, [EMIM][FSA] and [BMIM][TFSI] display checkerboard type arrangements on positively charged graphite and rubrene, respectively. Although the composite ions and the substrate differ from those in this study, it would be reasonable to assume that [EMIM][FSA] would similarly give the checkerboard arrangement on the C_9_-DNBDT-NW substrate. Combining the MD simulations and the present spectra, the molecular structure and arrangement on the organic semiconductor should be revealed, which is currently under investigation.

The structures of the interfacial ILs and their interaction with the organic semiconductor surface have strong effects on the device performance of OFETs. Recently, Okaue et al.^38^ reported carrier trapping dynamics at the interface in IL-gated EDL-OFETs. They reported an increase in the operation voltage (i.e., bias stress) of an IL-gated EDL-OFET using [EMIM][FSA] and the rubrene single crystal. The measurements of transfer curves, electrochemical impedance spectroscopy, atomic force microscopy, and MD simulations revealed that the bias stress originated from carrier trapping at the hole carrier-injected rubrene surface by the IL. As discussed in the next paragraph, the trap states of OFETs were studied by some groups using CMS. In this study, however, the IL-gated EDL-OFET consisting of [EMIM][FSA] and C_9_-DNBDT-NW, displayed no shift of transfer curves for 30 min as shown in Fig. S5. In addition, as described in Fig. 4, the spectral changes due to the applied gate voltage were reversible (i.e., there was no hysteresis). The transfer curve remained almost the same shape at the scan rate from 10 to 100 mV s^−1^. These results indicate that the fabricated EDL-OFETs are stable and that the effect of carrier trapping is negligible in the present case. This may be due to the weaker electrostatic interaction between the hole in C_9_-DNBDT-NW and [FSA]^−^ compared to that between the hole in rubrene and [FSA]^−^ because of the long alkyl chain of C_9_-DNBDT-NW. Recently, we are investigating the trapping mechanism using another organic semiconductor that electrostatically interacts with ILs more strongly by the EC-ATR-UV spectroscopy because it can directly measure the electronic absorption of both organic semiconductors and interfacial ILs. Related to the interaction between the organic semiconductor film and the ILs, the band bending between them and its modulation upon the voltage application are also points of interest. The present experimental data of the ILs/C_9_-DNBDT-NW system cannot access to these issues. However, there is a possibility that ILs/other organic semiconductors such as rubrene and fullerene, which do not have the insulating alkyl chains at the crystal surface with the thickness of ~1 nm like C_9_-DNBDT-NW will give some information due to the stronger interaction between the ILs and the organic semiconductors, leading to a discussion about the conduction mechanism in organic semiconductors.

Sirringhaus’s group reported drain voltage dependence of drain current and CMS of pentacene-based FETs at 100 K, and discussed trap states in OFETs^35^. The shallow trap decreased the drain current. The CMS detected absorption due to the shallow trap around 1.24 eV, and the absorbance decreased with increasing the drain voltage. Zhu’s group measured CMS of rubrene-based FETs in the IR region and revealed a discrepancy between the drain current and spectral changes, which was due to the trapping effects^26^. In this way, the trap states in OFETs influence both drain current and CMS spectra. Additionally, the carrier trapping resulted in hysteresis of transfer curve of OFETs^20,35^. On the other hand, in the present study, there was little hysteresis in both the transfer curve (Fig. 3a) and the spectral changes (Fig. 4). In addition, the EC-ATR-UV spectra showed no dependence on the drain voltage as shown in Fig. S6. These results also indicate that the trapping effect is small in ILs/C_9_-DNBDT-NW OFETs. Although the EC-ATR-UV spectroscopy did not detect the trapping states in organic semiconductors, it measured the absorbance and its Stark shift of the interfacial ILs on the organic semiconductors, which was its advantage compared to the reported drain current measurements and other spectroscopic techniques.

## Conclusion

In summary, IL-gated EDL-OFETs using C_9_-DNBDT-NW were successfully fabricated on the ATR prism. Whilst employing these as transistors, ATR-UV spectra were recorded using our newly developed spectroscopic system. In response to the applied gate voltage, the spectral peaks of the organic semiconductor shifted and bleached. This was correlated with the drain current. Such positive correlation among the applied gate voltage, drain current, and spectral changes corresponded with the previous reports utilizing CMS spectroscopies in the IR and visible regions. Our study revealed the dependence of the electrostatic interaction between the charged organic semiconductors and interfacial ILs, on the species of IL. In addition, EC-ATR-UV spectroscopy could measure the absorbance due to the ILs near the surface of the C_9_-DNBDT-NW film because of the short penetration depth and the strong ILs’ absorbance of the light in the UV region, which was a unique point of the new technique. Such a direct detection of the interfacial ILs will lead to the mechanism research of the EDL-OFTEs in combination with the simulations. The new system can access to the interfacial area between the various films and ILs. For example, the study of ILs/fullerene or metal electrode systems using the EC-ATR-UV spectroscopy is in progress. It is envisaged that this new method will be applied to other electrochemical devices such as organic thin-film solar cells, in which the interfacial region is crucial to their functioning.

## Materials and methods

### IL-gated EDL-OFET preparations

C_9_-DNBDT-NW films were fabricated on ATR sapphire prisms. The ATR prism (UV grade, purchased from Opt-line) was 9 mm by 8 mm with 2 mm thickness, and a fluororubber O-ring (3 mm diameter) was put in the center of the ATR prism. The measurement area was inside the O-ring, and the C_9_-DNBDT-NW film fully covered it. C_9_-DNBDT-NW allows for the production of inch size, single crystal-like thin films by a simple printing technique^22,23^. To this end, single crystal-like thin films with two molecular layers of C_9_-DNBDT-NW were grown on glass substrates, which had been pretreated with UV-ozone, from a 0.02 wt% 3-chlorothiophene solution using continuous edge-casting, according to literature procedures^23^. The substrate containing the C_9_-DNBDT-NW film was then inverted on the ATR sapphire prism^23^. A few droplets of ultrapure water were applied near the point of contact between the two substrates (i.e., glass and ATR sapphire prism). Finally, the glass substrate was carefully taken away to complete the transfer. Prior to the electrode deposition described below, the sapphire prisms with the transferred C_9_-DNBDT-NW film were thermally annealed at 80 °C for 10 h in a vacuum oven. It was confirmed that the crystallinity of the transferred C_9_-DNBDT-NW film was remained using X-ray diffraction^23^.

Gold (Au) films were evaporated to a thickness of ~40 nm on the C_9_-DNBDT-NW films as the source and drain electrodes (working electrodes) on both sides of the ATR prism with 1.6 mm channel space (Fig. 1b). ILs were cast on the prepared film, and a Pt coil (0.1 mm diameter, ~1 cm length) and a Pt wire (0.1 mm diameter, ~10 cm^2^) were placed in the ILs as pseudo counter and reference (gate) electrodes, respectively. The electrodes were then connected to a bipotentiostat (Autolab PGSTST128N combined with BA and FRA32 module, Metrohm). Subsequently, 1 mL of ILs, [EMIM][FSA] and [TMPA][TFSA], were cast on the C_9_-DNBDT-NW films. They were purchased from Kanto Kagaku Co., Inc., and they were heated under vacuum (∼80 °C, 10^−2^ Pa, 20 h) immediately prior to the experiments. Even though it is known that the Pt pseudo electrode cannot determine the accurate potential^41^, we adopted them because of its ease of handling.

### EC-ATR-UV spectroscopy measurements

The prepared device on the sapphire prism was placed on the custom-made spectrometer, which consisted of the UV–vis–NIR spectrophotometer (V-770, JASCO) and an original ATR unit. The light sources were a deuterium lamp (<250 nm) and a halogen lamp (≥250 nm). The incident light passed through a monochromator and irradiated into the ATR prism. In order to cancel out temporal intensity fluctuations, a double beam method was adopted. That is to say, the incident light from the monochromator was spitted into reference and sample beams, and the reference beam was always irradiated into a bare ATR prism. The reflected light was detected by a photomultiplier. The incident angle was set to 70°, and the penetration depth was approximately 50 nm or less in the measured wavelength region (200–500 nm). The typical measurement time for obtaining one spectrum (200–500 nm) was 3 min (100 nm/min). In the EC spectral measurements, the drain voltage *V*_D_ was maintained at −100 mV, whereas the gate voltage *V*_G_ has adjusted in 100 mV intervals between 0 and −1000 mV. For comparison, the transmission spectra of the C_9_-DNBDT-NW/dichloromethane solution and the C_9_-DNBDT-NW film on the sapphire substrate were measured using the UV–vis–NIR spectrophotometer. The original ATR unit and the commercial transmission unit could be easily exchanged. Experimentally obtained spectra were analyzed by DFT and TD-DFT calculations. Ground-state geometry optimizations were performed using the B3LYP method with the 6–31G+ (d,p) basis set. Subsequently, vertical transition energies were calculated using the TD-CAM (Coulomb-attenuating method)-B3LYP technique with the 6–31+G(d,p) basis set. All calculations were performed using the Gaussian 16 suite of programs^42^.

## Supplementary information

Supplementary Materials

## Data Availability

All data needed to evaluate the conclusions in the paper are present in the paper and/or the Supplementary Materials. Additional data related to this paper may be requested from the authors.
