# Supplementary material for: Electronic excitation spectra of organic semiconductor/ionic liquid interface by electrochemical attenuated total reflectance spectroscopy
Source: Commun Chem. 2021 Jun 11;4:88. doi: 10.1038/s42004-021-00525-y (PMC9814848; doi:10.1038/s42004-021-00525-y)
Supplement: Supplementary file 1 — Supplementary Materials [file 42004_2021_525_MOESM1_ESM.pdf]

Supplementary Materials for

Electronic excitation spectra of organic semiconductor/ionic liquid interface by electrochemical attenuated total reflectance spectroscopy

I. Tanabe,[1]* I. Imoto,[1] D. Okaue,[1] M. Imai,[1] S. Kumagai,[2] T. Makita,[2] M. Mitani,[2] T. Okamoto,[2] J. Takeya,[2] K. Fukui[1]*

1 Graduate School of Engineering Science, Osaka University, 1-3 Machikaneyama-cho, Toyonaka, Osaka 560-8531, Japan
2 Graduate School of Frontier Sciences, The University of Tokyo, Kashiwa, Chiba 277-8561, Japan

*itanabe@chem.es.osaka-u.ac.jp (I.T.)
*kfukui@chem.es.osaka-u.ac.jp (K.F.)

This PDF file includes:

Table S1. Summary of calculated vertical transitions and their main assignments.
Fig. S1. Quantum chemical calculation of a C_9-DNBDT-NW molecule.
Fig. S2. Molecular orientation effect on a transmission spectrum of a C_9-DNBDT-NW film.
Section S1. Estimation of carrier mobility.
Fig. S3. Impedance analysis of C_9-DNBDT-NW OFETs.
Fig. S4. Comparison with transmission spectroscopy.
Fig. S5. An effect of carrier trapping.
Fig. S6. Drain voltage dependence.

Table S1. Summary of calculated vertical transitions and their main assignments. Calculated transition energies, oscillator strengths, main initial states, and main final states of a C_9-DNBDT-NW molecule. Polarizable continuum model (PCM) was used to account for the solvent (dichloromethane) effect.

Wavelength / nm	Energy / eV	Oscillator strength	Main initial states	Main final states
414.9	2.988	0.2128	HOMO	LUMO
364.7	3.400	1.1871	HOMO−2	LUMO
309.7	4.004	0.8334	HOMO	LUMO+2
299.4	4.141	0.5339	HOMO−1	LUMO+1
287.3	4.316	0.1435	HOMO	LUMO+3
270.3	4.586	0.1883	HOMO−2	LUMO+2
264.2	4.693	0.8943	HOMO−2	LUMO+3
247.8	5.003	0.1174	HOMO	LUMO+7
241.9	5.126	0.9919	HOMO−1	LUMO+4

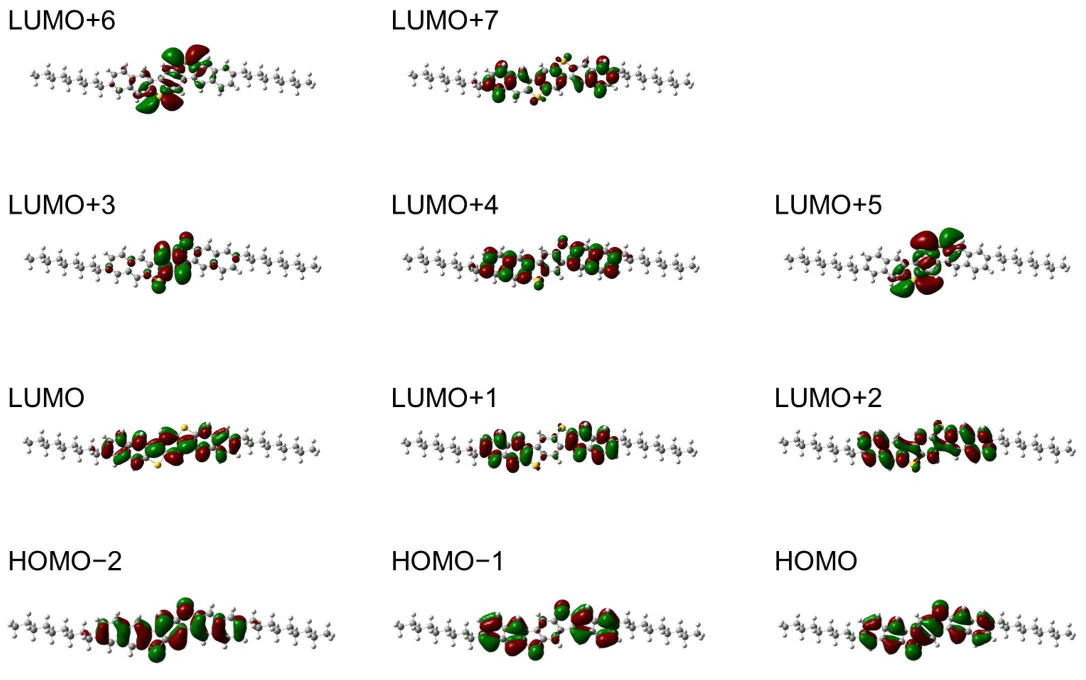

Fig. S1. Quantum chemical calculation of a C9-DNBDT-NW molecule. Calculated molecular orbitals under identical conditions with an isovalue of 0.02.

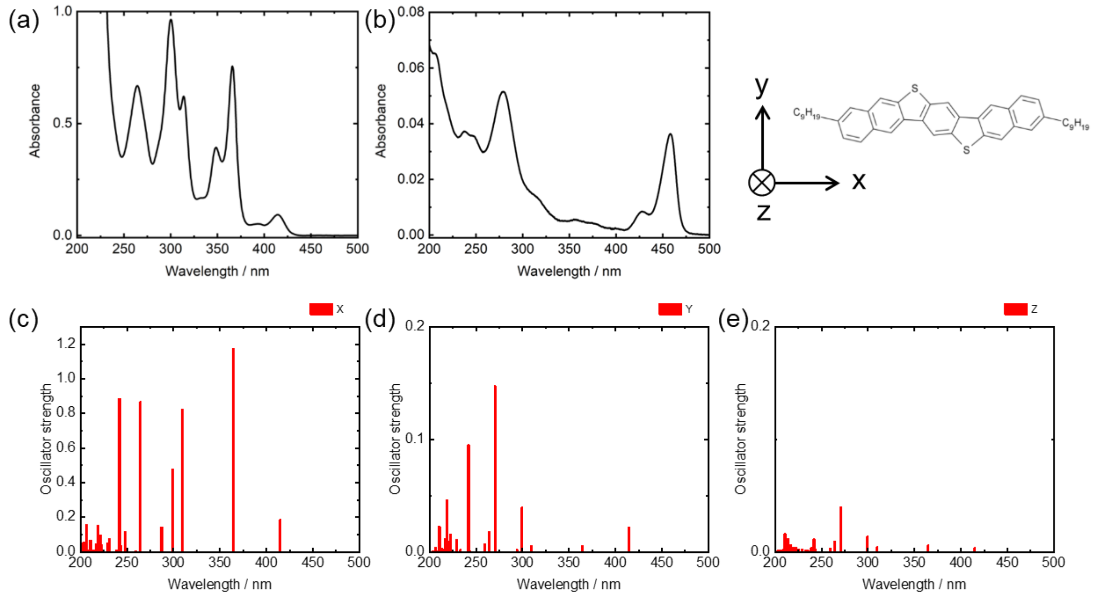

Fig. S2. Molecular orientation effect on a transmission spectrum of a C9-DNBDT-NW film. (a,b) Transmission spectra of (a) C_9-DNBDT-NW/dichloromethane solution (same data as Figure 2a in main text) and (b) C_9-DNBDT-NW film on a sapphire substrate (same data as Figure 3 in main text). (c-e) Calculated vertical transitions along the (c) x-, (d) y-, and (e) z-axis of the C_9-DNBDT-NW molecule.

According to the TD-DFT calculations (Fig. S2c-d), the vertical transitions along the x-axis (i.e. a-axis of a C_9-DNBDT-NW molecule) are much larger than those along y- and z-axes. The transmission spectrum of the C_9-DNBDT-NW film (Fig. S2b) was closely reproduced by using calculated vertical transitions along the y-axis (Fig. S2d). This is because the C_9-DNBDT-NW molecules are oriented as described in Fig. 1b in the main text. The elsectric field vibration direction of the incident light for the transmission spectra is perpendicular to the x-axis and parallel to the y- and z-axes. The oscillator strength along the z-axis is much smaller than that along the y-axis. Therefore, the transmission spectrum of the C_9-DNBDT-NW film strongly reflects the electronic transition along the y-axis.

Section S1. Estimation of carrier mobility.

Based on the transfer curve of the fabricated IL-gated EDL-OFET (Figure 4a in the main text), its mobility μ in the linear region was estimated as follows:

$$\mu = \frac{L \cdot \alpha}{W \cdot C_{\mathrm{EDL}} \cdot V_{\mathrm{D}}} \qquad \text{(S1)},$$

where L, W, C_{EDL}, V_{D}, and α represent the channel length, channel width, capacitance of EDL per unit area, drain voltage, and slope of the transfer curve, respectively. In the present case, L and W were about 1.6 mm and 8.0 mm, respective. The value of C_{EDL} was estimated at ~2.0 μF by electrochemical impedance spectroscopy (10-10^6 Hz) using the previously reported equivalent circuit shown in Fig. S3 (*38*). In Fig. S3a, C_{C} and R_{C} are the contact capacitance and the resistance between rubrene and the Au (source and drain) electrodes, respectively. C_{ch} and R_{ch} are the capacitance and resistance of the channel, respectively. C_{EDL} is the CPE of EDL between the IL and C_9-DNBDT-NW interface. R_{s} is the solution resistance of IL. V_{D} was set at 100 mV. According to Figure 4a in the main text, the estimated mobility (μ) using the value of the slope (α) in the 800-100 mV region was 4.9 $cm^2\ V^{-1}\ s^{-1}$, which corresponded with the reported mobility of SiO_2-gated OFET (~4-13 $cm^2\ V^{-1}\ s^{-1}$) (*21*).

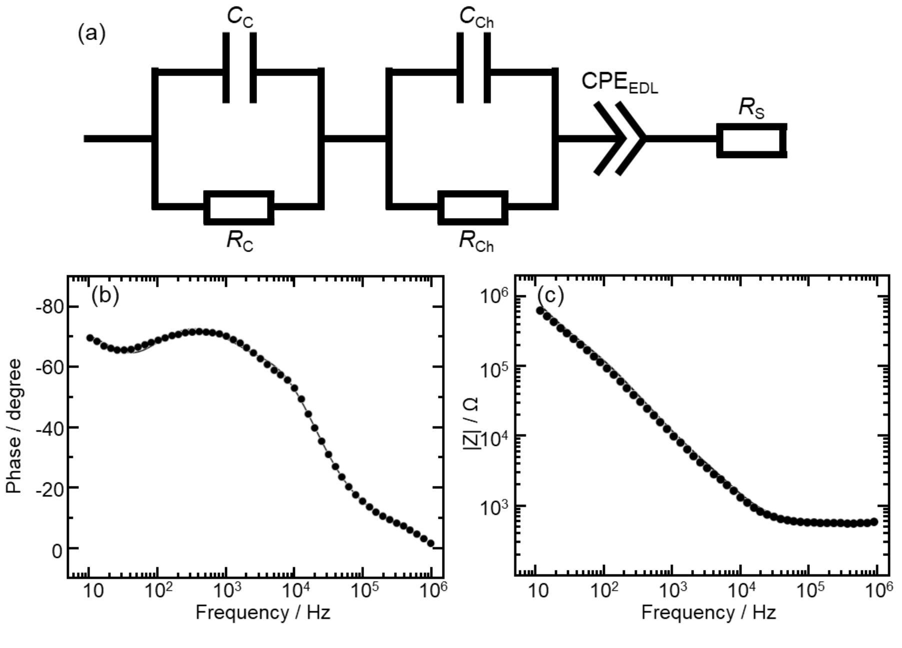

Fig. S3. Impedance analysis of C9-DNBDT-NW OFETs. (a) Equivalent circuit for the impedance analysis of EDL-OFET. (b) Phase angle and (c) |Z| vs. frequency. The dots are the measured results, and the solid lines are their fittings.

Section S2. Estimation of penetration depth.

The penetration depth d_p is calculated as follows (N. J. Harrick, *Phys. Rev. Lett.* **4**, 224 (1960)), where λ, θ, n_1, and n_2 are incident light wavelength, incident light angle, refractive indicis of ATR prism and sample, respectively.

$$d_\mathrm{p} = \frac{\lambda}{2\pi n_1\sqrt{\sin\theta - (n_2\,/\,n_1)^2}} \quad \text{(S2)},$$

In this study, the measurement wavelength was 200-500 nm, and the incident angle was set at 70°. The penetration depth around 210 nm is particularly important to discuss interfacial region between the C_9-DNBDT-NW films and ILs. This is because [EMIM][FSA] has strong absorbance around 210 nm as shown in Fig. 5a and Fig. 6 in the main text. When the refractive indices of the sapphire ATR prism (n_1) and the organic semiconductor (n_2) are assumed 1.8 and 1.5, respectively, the penetration depth is calculated ~40 nm at 210 nm. The accurate refractive index and its wavelength dependence of a C_9-DNBDT-NW film in the UV region are unclear, and thus, the value of n_2 (1.5) was used the value measured by a refractive index sensor (PAL-RI, Atago Co.). As shown in Fig. 2c, the absorbance of the C_9-DNBDT-NW film at 210 nm was ~0.06 (i.e. transmittance was ~87%). According to these calculations, the present EC-ATR-UV technique detects the absorbance of ILs existing in the range of ~40 nm from the C_9-DNBDT-NW film surface. More accurate estimation of the penetration depth and its control by the incident angle modulation are important issues for the further development of the EC-ATR-UV spectroscopy.

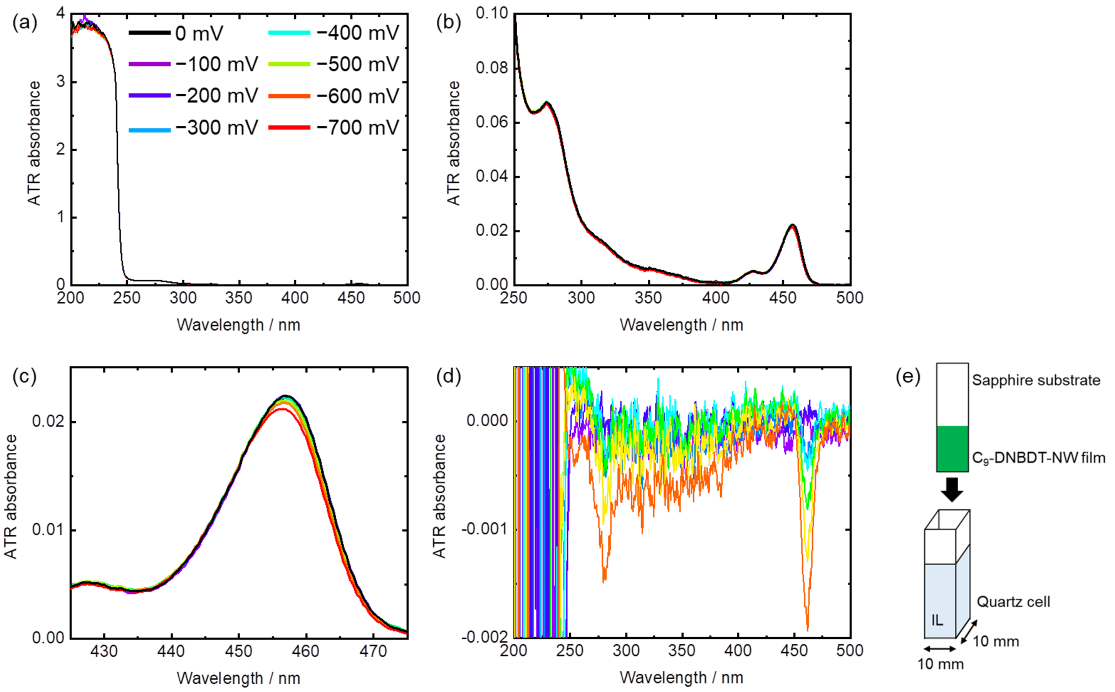

Fig. S4. Comparison with transmission spectroscopy. (a-c) Transmission and (d) difference spectra of a C_9-DNBDT-NW film on a sapphire substrate with [EMIM][FSA] using a 10 mm quartz cell for the (a) 200–500 nm, (b) 250–500 nm, and (c) 425–475 nm regions under the gate voltage application from 0 mV to +700 mV. (e) Schematic illustration of a setup for a transmission measurement.

As shown in Fig. S4a and S4b, transmission spectra have a strong absorbance below 260 nm due to [EMIM][FSA]. Spectral changes upon the gate voltage application in the 425-475 nm region (Fig. S4c) were similar to those in Fig. 3c in the main text. However, the spectral changes for the IL could not be investigated by transmission spectroscopy because of the spectral saturation (Fig. S4d). Fig. S4e shows the schematic illustration of the setup for the transmission measurement.

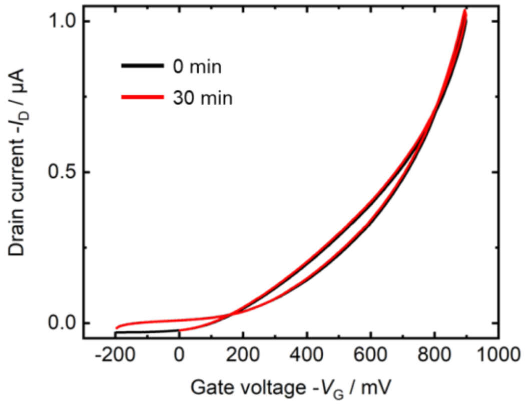

Fig. S5. An effect of carrier trapping. Transfer curves of the fabricated device measured at the drain voltage V_D = -100 mV and scan rate = 20 mV s^{-1}.

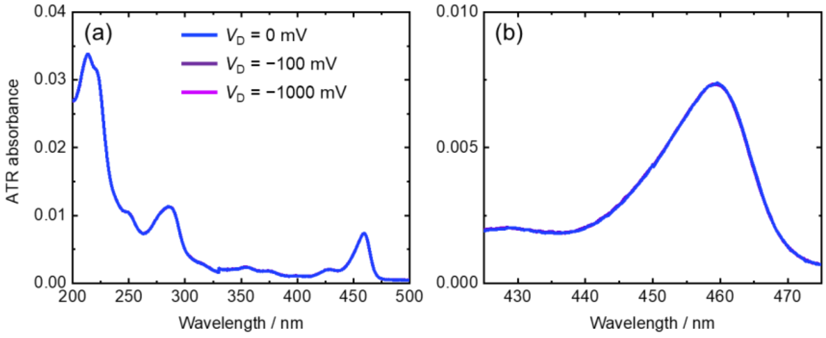

Fig. S6. Drain voltage dependence. ATR spectra in (a) 200-500 nm and (b) 425-475 nm of a C_9-DNBDT-NW film with [EMIM][FSA] on a sapphire substrate at V_G = -1000 mV and V_D = 0, -100, -1000 mV.
